# Human Metapneumovirus Infection in Chimpanzees, United States

**DOI:** 10.3201/eid2012.140408

**Published:** 2014-12

**Authors:** Owen M. Slater, Karen A. Terio, Yange Zhang, Dean D. Erdman, Eileen Schneider, Jane M. Kuypers, Steven M. Wolinsky, Kevin J. Kunstman, Jennifer Kunstman, Michael J. Kinsel, Kathryn C. Gamble

**Affiliations:** Lincoln Park Zoo, Chicago, Illinois, USA (O.M. Slater, K.C. Gamble);; University of Illinois, Maywood, Illinois, USA (K.A. Terio, M.J. Kinsel);; Battelle, Atlanta, Georgia, USA (Y. Zhang);; Centers for Disease Control and Prevention, Atlanta (D.D. Erdman, E. Schneider);; University of Washington, Seattle, Washington, USA (J.M. Kuypers);; Northwestern University, Chicago (S.M. Wolinsky, K.J. Kunstman, J. Kunstman)

**Keywords:** human metapneumovirus, viruses, chimpanzees, infectious disease, zoonoses, United States

## Abstract

Zoonotic disease transmission and infections are of particular concern for humans and closely related great apes. In 2009, an outbreak of human metapneumovirus infection was associated with the death of a captive chimpanzee in Chicago, Illinois, USA. Biosecurity and surveillance for this virus in captive great ape populations should be considered.

Zoological facilities in North America house endangered species of great apes with annual visitation rates of >100 million persons (*1*). Because humans and great apes are related genetically, interspecies transmission of infectious pathogens is a concern. Consequently, procedures are instituted to limit the potential spread of infectious pathogens (*2*).

Reports of outbreaks of human metapneumovirus (HMPV) infection with respiratory symptoms have been documented in wild great ape populations (*3**–**5*). All of these outbreaks have been attributed to exposure to humans because of the suspected HMPV-negative status of these populations. However, disease caused by HMPV has not been previously documented in North American zoo populations, despite the close proximity of humans and great apes. We report an outbreak of HMPV infection in 2009 in a troop of previously HPMV-negative chimpanzees (*Pan troglodytes*) in Chicago, Illinois, USA, that resulted in 1 death and an illness rate of 100%.

## The Study

Two chimpanzee and 2 western lowland gorilla (*Gorilla gorilla gorilla*) troops were housed in separate areas within 1 building with shared airspace at a zoological facility in Chicago, Illinois, USA. Animals had periodic contact with keepers during daily feeding, cage cleaning, and training sessions. Biosecurity for staff in the great ape area included wearing gloves, dedicated footwear and clothing, handwashing, and use of footbaths when entering and exiting the facility and during movement between troops. Staff members were required to notify management if they had a confirmed or suspected respiratory infection so that they were removed from direct interaction with animals. No outside personnel were allowed direct contact; indirect contact was considered rare and only possible if visitors tossed objects into outdoor enclosures.

**Figure 1:**
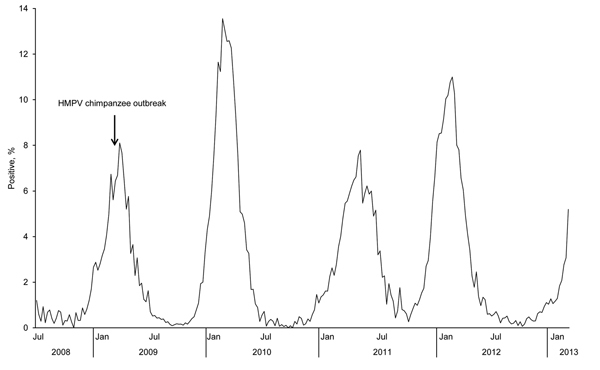
Percentage of human metapneumovirus (HMPV)–positive test results, by week of report, National Respiratory and Enteric Virus Surveillance System, United States, July 2008–January 2013.

Within 1 week before the outbreak in chimpanzees, staff members at the great ape facility had respiratory disease (coughing and nasal discharge), which coincided with peak HMPV season in the United States (Figure 1). The affected chimpanzee troop consisted of 7 chimpanzees; the initial clinical sign (coughing) on March 18, 2009, was observed in 1 adult female (Table).

**Table:** Serologic results for IgG against HMPV in chimpanzees and gorillas at the incident zoological facility, United States, 1994–2012*

Great ape	Age at outbreak, y†	Arrived at facility	Year																				
			1994	1995	1996	1997	1998	1999	2000	2001	2002	2003	2004	2005	2006	2007	2008	2009		2010		2011	2012
Chimpanzee troop 1																							
AM	18.3	2004	NT	NT	NT	NT	NT	NT	NT	NT	NT	NT	NT	NT	N	NT	N	N‡	P			NT	P
AF1	24.6	2004	NT	NT	NT	NT	NT	NT	NT	NT	NT	NT	NT	NT	N	NT	N	NT	NT			NT	P
AF2	18.6	2004	NT	NT	NT	NT	NT	NT	NT	NT	NT	NT	NT	NT	N	NT	N	NT	NT			P	NT
AF3	15.2	2004	NT	NT	NT	NT	NT	NT	NT	NT	NT	NT	NT	NT	N	NT	NT N	NT	NT			NT	P
SAM1	9.2	2004	NT	NT	NT	NT	NT	NT	NT	NT	NT	NT	NT	NT	N	NT	N	N§	NT			NT	NT
SAM2	10.1	2004	NT	NT	NT	NT	NT	NT	NT	NT	NT	NT	N	NT	N	NT	N	NT	NT			P	NT
SAF	9.5	2004	NT	NT	NT	NT	NT	NT	NT	NT	NT	NT	NT	NT	N	NT	N	NT	NT			P	NT
Chimpanzee troop 2																							
AM	50.8	1959	NT	NT	P	NT	NT	P	NT	P	P	NT	NT	P	NT	NT	NT	NT	NT		NT		P
AF1	43.4	1965	NT	P	P	NT	NT	P	P	P	P	NT	P	NT	P	NT	P	NT	NT		NT		P
AF2	44.2	1971	P	NT	P	NT	NT	P	NT	P	P	NT	NT	NT	P	NT	P	NT	NT		NT		NT
AF3	28.6	1980	P	NT	P	NT	NT	P	P	NT	P	NT	NT	NT	P	NT	P	P	NT		NT		P
AF4	43.8	1970	P	NT	P	NT	NT	NT	P	P	P	NT	NT	NT	P	P	NT	NT	NT		NT		NT
Gorilla troop 1																							
AM	20.1	1998	NT	NT	NT	NT	N	NT	NT	P	NT	NT	NT	P	NT	NT	P	NT	P		NT		NT
AF1	31.2	1978	NT	P	P	NT	NT	NT	NT	P	NT	NT	NT	NT	P	NT	P	NT	NT		NT		NT
AF2	20.2	1989	NT	NT	NT	NT	N	NT	P	NT	NT	NT	NT	NT	P	NT	P	P	NT		NT		NT
AF3	12.8	1996	NT	NT	NT	NT	NT	NT	NT	P	NT	NT	NT	NT	P	NT	P	P	NT		NT		NT
JM	3.6	2005	NT	NT	NT	NT	NT	NT	NT	NT	NT	NT	NT	NT	NT	NT	N	NT	NT		P		NT
Gorilla troop 2																							
AM	28.9	1980	NT	NT	NT	NT	NT	NT	NT	NT	P	NT	NT	NT	P	NT	P	NT	NT		NT		P
AF1	21.7	1987	NT	NT	N	N	NT	N	NT	NT	P	NT	NT	P	NT	NT	P	NT	NT		NT		P
AF2	18.5	1990	P	NT	NT	P	NT	NT	P	NT	P	NT	NT	NT	NT	P	P	NT	NT		P		NT
AF3	16.9	1992	NT	N	NT	N	N	NT	P	NT	P	NT	NT	NT	P	NT	P	P	NT		NT		NT
AF4	12.5	1996	NT	NT	NT	NT	NT	NT	NT	P	NT	NT	NT	P	NT	P	NT	P	NT		NT		NT
JM1	5.3	2004	NT	NT	NT	NT	NT	NT	NT	NT	NT	NT	NT	N	N	NT	N	NT	NT		NT		NT

*HPMV, human metapneumovirus; AM, adult male; NT, not tested (animal was not present at the facility; not born; died; or sampling was not performed that year); N, HMPV IgG seronegative; P, HPMV IgG HMPV seropositive; AF, adult female; SAM, subadult male; SAF, subadult female; JM, juvenile male. Gray shading indicates seroconversion or ≥4-fold increase in HMPV IgG titer, and black shading indicates death caused by HMPV. †Day of symptom onset for HMPV outbreak was March 18, 2009.  ‡Serum collected January 2009 and antibody negative.  §Serum collected postoutbreak on March 23 and no IgG against HMPV was detected.

Within 96 hours, all 7 chimpanzees had moderate-to-severe respiratory disease (≥2 characteristic clinical signs), and their intake of oral fluids was increased. One juvenile male with pectus excavatum had marked mucopurulent rhinorrhea, coughing, lethargy, tachypnea, dyspnea, and partial anorexia; he was also given an intramuscular broad-spectrum antimicrobial drug (ceftiofur, 25 mg/kg). Within 24 hours, the condition of this animal worsened, which necessitated sedation for rehydration and diagnostic assessment. Radiographs showed marked bronchointerstitial pneumonia. An additional antimicrobial drug (cefazolin, 25 mg/kg) and fluids (0.9% NaCl, 40 mL/kg/h) were administered, but the animal died the next morning.

The remaining animals in the troop were given antimicrobial drugs (cefazolin, 25 mg/kg; enrofloxacin, 5 mg/kg) and anti-inflammatory medication (flunixin meglumine, 0.25 mg/kg). Within 48 hours, all animals showed mild improvement. Antimicrobial drugs were given for 10 days. Fifteen days post-onset, clinical resolution had occurred. The other troops showed no signs of respiratory disease.

**Figure 2:**
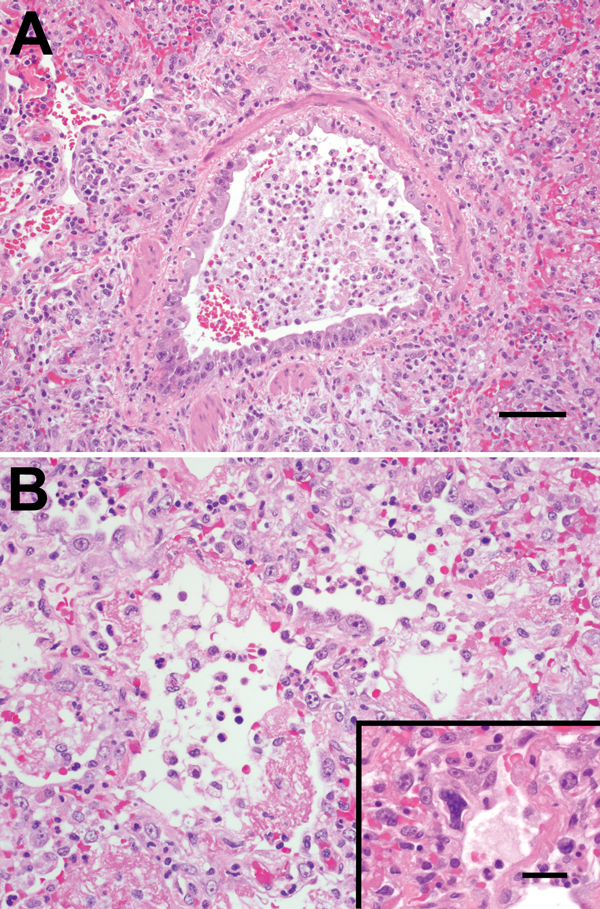
A) Bronchiolar epithelium of chimpanzees infected with human metapneumovirus, United States, 2009, showing cell variation from attenuated to piled and disorganized. Epithelial cells lack cilia, and lumens contain foamy macrophages, neutrophils, and hemorrhage. Adjacent air spaces are filled with similar inflammatory cells. Scale bar = 70 μm. B) Alveoli lined with plump type II pneumocytes and fibrin. Inset: Rare, deeply basophilic, smudged nuclei are present in some areas. Scale bar = 20 μm. Hematoxylin and eosin stain.

Necropsy of the animal that died showed that the lungs were firm, had not collapsed, and surrounding airways were mottled red-to-purple. Histologic analysis showed that lesions were similar to those observed in humans and indicated necrotizing bronchointerstitial pneumonia with type II pneumocyte hyperplasia, abundant fibrin, and streaming mucus in airways. Cilia were absent from many bronchial epithelial cells, and rare epithelial cells lacked any identifiable cytoplasmic membrane or nuclear structure, suggestive of the smudge cells commonly found in human HMPV infection (*6*) (Figure 2).

Only rare gram-positive cocci were observed, and the lack of extensive suppurative inflammation suggested that these findings were not a major contributing factor. Bacterial lung tissue cultures were negative. Lung tissue was screened for viral respiratory pathogens by real time reverse transcription PCR by using published methods (*7*). Sections were positive for HMPV and negative for adenoviruses, coronaviruses, influenza viruses A and B, human parainfluenza viruses 1–4, bocavirus, rhinovirus, and respiratory syncytial virus.

The complete HMPV nucleoprotein gene was sequenced and deposited in GenBank (accession no. KF891365). The HMPV strain sequence showed 99% nucleotide identity with group A, subgroup A2 reference strain CAN97–83 (accession no. AY297749.1).

To assess troop HMPV exposure, we conducted a serologic study by measuring IgG against HMPV in available serum samples collected at various times before and after the outbreak (Table) (*8*). Serum samples were usually collected opportunistically, often not during clinical illness. Unlike samples from other troops, serum samples from chimpanzee troop 1 showed that these animals were seronegative for HMPV before the outbreak; 100% seroconversion was observed 1–3 years later. Serum samples obtained before 2009 from the other troops had stable levels of IgG against HMPV, and these troops had experienced 12 episodes in which a ≥4-fold increase in titer or seroconversion were noted, indicating exposure within the testing interval. Overall, HMPV seroprevalence in the year before the outbreak for the chimpanzee and gorilla troops were 42% and 75%, respectively. Seroprevelance 3 years post-outbreak was 100% and 92%, respectively.

## Conclusions

We report an outbreak of HMPV infection producing illness and death in chimpanzees in a North American zoo. Although the human source of the infection remains unknown, staff members in the great ape area had respiratory disease just before the outbreak in chimpanzees. Serologic testing of staff for respiratory viruses could not be performed. Post-outbreak, in addition to biosecurity measures already in place, all staff working with primates have been required to wear facemasks during direct primate interactions.

Unlike this situation, chimpanzees experimentally infected with HMPV have shown only mild cold-like clinical signs in seronegative animals and no clinical signs in seroconverted animals (*9*). Seroconverted, naturally infected, captive-bred chimpanzees represented 61% of the laboratory population, which demonstrated that captive animals are readily infected with HMPV (*9*). Worldwide, nearly 100% of the human population has seroconverted to HMPV by 10 years of age, and most illness and death occurs in young, elderly, and immunocompromised persons, although persons in any age group can become infected (*10**,**11*). Immunity is transient, and reinfections with the same or different strains are common, but illness is reduced (*10**,**11*). Recent evidence suggests these findings are also found in captive and wild chimpanzees (*9**,**12*) and other primates (*13*). In this instance, the congenital thoracic defect may have affected the ability of the chimpanzee to survive. In contrast to other cases in which apes were infected with *Streptococcus pneumoniae* (*3*–*5*), only rare lung bacteria were observed histologically and lung bacterial cultures were negative. These findings, in combination with the absence of major suppurative inflammation, suggest that HMPV was the primary pathogen.

Given the ubiquitous nature of this human virus in North America and the frequency with which it infects humans, it is notable that all members of the affected chimpanzee troop were born in captivity and had contact with humans throughout their lives, yet still remained negative for this virus until March 2009. Because HMPV is a recently discovered virus that closely resembles other respiratory viruses in its clinical course and few animal facilities specifically test for it, the degree of illness associated with this disease in the captive great ape population is unknown. Therefore, enhanced biosecurity and disease surveillance measures for HMPV should be considered for great apes. In addition to the commonly tested viral respiratory pathogens of great apes, surveillance for HMPV by serologic analysis at quarantine or preventative medical examinations would provide additional benefits. These procedures would enable management to tailor biosecurity protocols and procedures to limit the risk for exposure of HMPV-negative animals or troops, particularly during the height of the HMPV season.
